# Predicting outcomes for recurrent hepatocellular carcinoma within Milan criteria after complete radiofrequency ablation

**DOI:** 10.1371/journal.pone.0242113

**Published:** 2020-11-10

**Authors:** Hsin-Yeh Chen, Sheng-Nan Lu, Chao-Hung Hung, Jing-Houng Wang, Chien-Hung Chen, Yi-Hao Yen, Yuan-Hung Kuo, Kwong-Ming Kee

**Affiliations:** Division of Hepatogastroenterology, Department of Internal Medicine, Kaohsiung Chang Gung Memorial Hospital, and Chang Gung University College of Medicine, Kaohsiung City, Taiwan; Nihon University School of Medicine, JAPAN

## Abstract

**Background:**

Intrahepatic distant recurrence (IDR) is a significant problem for patients who have undergone radiofrequency ablation (RFA) for hepatocellular carcinoma (HCC). The objective of the study was to investigate risk factors and to predict outcomes of recurrent IDR within Milan criteria after complete RFA for primary early-stage HCC.

**Method:**

This retrospective study reviewed 449 patients with intrahepatic distant recurrent HCC after complete RFA for early-stage HCC. After excluding 100 patients who were beyond Milan criteria, with incomplete lab data, or had follow-up less than three months, a total of 349 patient cases were compiled and their baseline characteristics, further treatment modalities after tumor recurrence and survival were analyzed.

**Results:**

After a median follow-up of 36.2 months, 92 patients had expired. The majority of patients were male (59.9%) with a median age of 64.3 years (range:38–88). The cumulative 5-year overall survival (OS) rates after treatment for recurrent HCC was 67.2%. On multivariate analysis, end-stage renal disease(Hazard ratio (H.R.) = 2.33, p = 0.021), m-ALBI grade 2a (H.R. = 2.86, p = 0.003) and m-ALBI grades 2b or 3 (H.R. = 2.30, p = 0.009), APRI greater than 1 (H.R. = 1.92, p = 0.036) and 2^nd^ recurrence occurring within 1 year (H.R. = 2.69, p<0.001) were significantly associated with worse survival. The cumulative 5-year 2^nd^ recurrence rate was 87.4%. On multivariate analysis, male gender (H.R. = 1.47, p = 0.01), age greater than 65 years (H.R. = 1.72, p<0.001), an alpha fetoprotein level greater than 20ng/ml (H.R. = 1.41, p = 0.016), surgical treatment for recurrent HCC (H.R. = 0.25, p = 0.007), tumor number greater than 1 (H.R. = 1.35, p = 0.046), and IDR developing within 2 years (H.R. = 1.67, p = 0.001) were prognostic factors for 2^nd^ recurrence.

**Conclusion:**

Our study suggested that presence of end-stage renal disease, m-ALBI grades 2 and 3, APRI >1 and time to 2^nd^ HCC recurrence were all associated with overall survival while the 2^nd^ HCC recurrence was associated with male gender, age ≥65 years, α-fetoprotein level >20 ng/mL, non-surgical therapy, time to IDR, and tumor number> 1.

## Introduction

Hepatocellular carcinoma (HCC) is the fourth most common cause of cancer-associated mortality globally and the third most common in Taiwan [1–3]. Ultrasonographic surveillance of patients with chronic liver disease provides the opportunity to detect early-stage HCC eligible for curative treatments, such as liver transplantation, tumor resection, and tumor local ablation. However, liver transplantation is limited by organ donor shortage and only 20% of patients are considered as candidates for curative hepatectomy [4]. Radiofrequency ablation (RFA), an effective local treatment, is recommended in patients with Barcelona Clinic Liver Cancer (BCLC) stages 0 and A HCC who are not fit for surgery [5].

Outcomes following RFA are comparable to surgical resection, especially for lesions smaller than 3 cm. The 5-year overall survival (OS) of RFA has been reported as 40.1–86% [6–9], but recurrence after ablation of early-stage HCC occurs in up to 60–85% of patients by 5 years [7, 9, 10]. Factors associated with OS have been studied over the past decade. Shiina et al. demonstrated OS was influenced by age, antibody to hepatitis C virus (anti-HCV), Child-Pugh class, tumor size, tumor number, serum level of alpha fetoprotein (AFP), and serum des-γ-carboxy-prothrombin (DCP) level [11]. As liver function reserve is critical for treatment of HCC and affecting OS, Child-Pugh classification has been used widely in prediction models. Newer grading systems for liver function reserve and fibrosis like albumin-bilirubin (ALBI) grade, modified albumin-bilirubin (m-ALBI) grade and aminotransferase-to-platelet ratio index (APRI) have been reported to be predictors of HCC [12–16].

To date, most studies predicting outcomes of patients after RFA utilize data at the time of initial diagnosis, yet with the increasing age of patients receiving treatment for malignancies, either progressive hepatic dysfunction or multiple comorbid disease such as diabetes or end-stage renal disease (ESRD) might be present when HCC recurrence takes place, and which might affect survival. It is crucial to identify prognostic factors prior to subsequent treatment for recurrent HCC, but most studies regarding prognostic factor for survival after HCC recurrence carried out hepatic resection for primary HCC [17, 18]. The aim of our study was to investigate factors associated with survival and 2nd recurrence when intrahepatic distant recurrence (IDR) after RFA has occurred, and to evaluate stratification ability and prognostic predictive value of modified-ALBI grade and APRI in such a cohort.

## Material and method

### Patients

**Fig 1:**
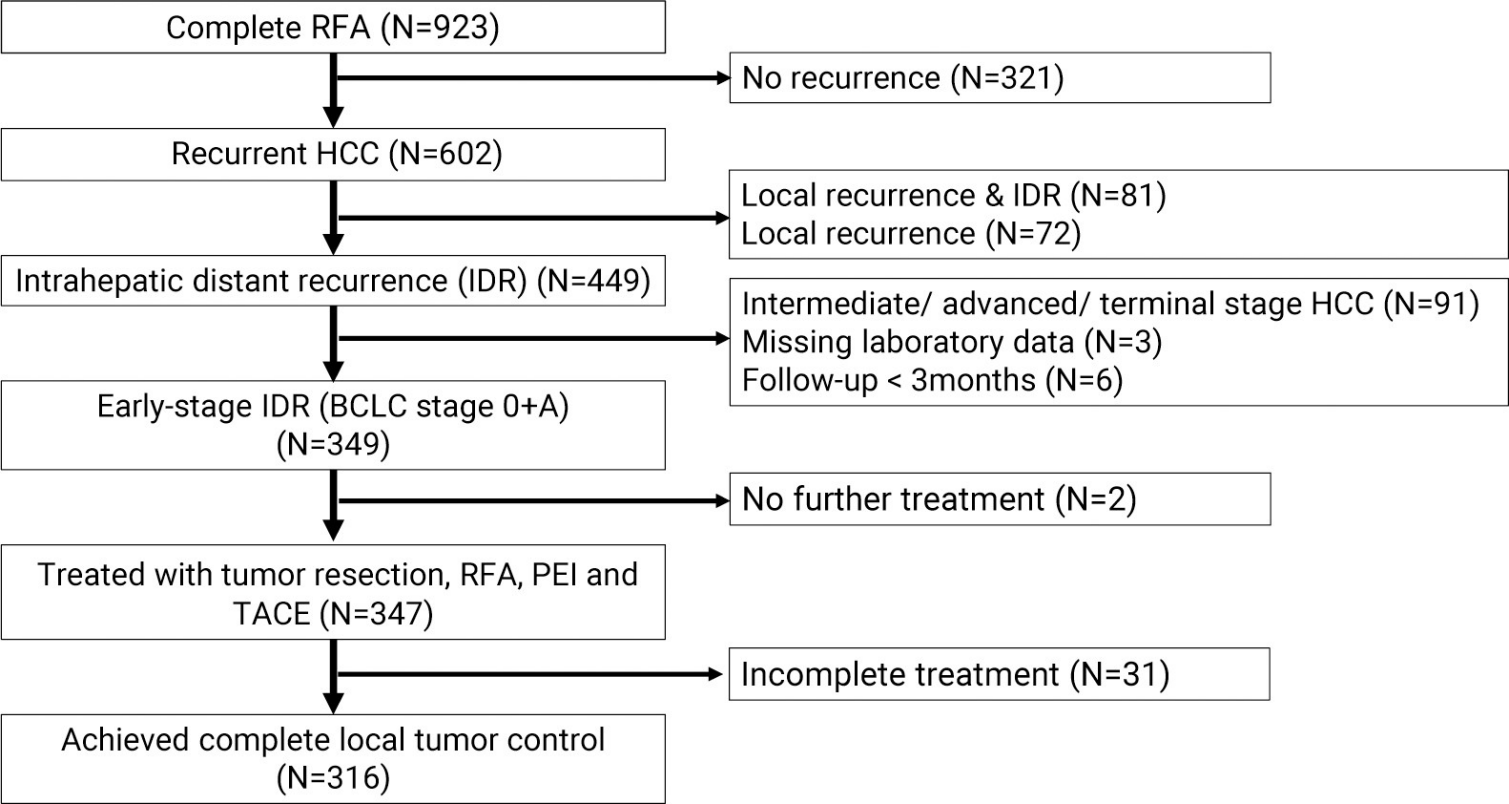
Patient flow chart. The chart included all patients with intrahepatic distant recurrence HCC in this study. HCC, hepatocellular carcinoma; IDR, intrahepatic distant recurrence; BCLC, Barcelona clinic liver cancer; RFA, radiofrequency ablation; PEI, Percutaneous ethanol injection; TACE, Transcatheter arterial chemoembolization.

In this retrospective cohort study, we reviewed 923 patients who received RFA for BCLC early-stage HCC as initial treatment and achieved complete ablation in Kaohsiung Chang Gang Memorial Hospital from June 2003 to September 2018 (Fig 1). A total of 349 patients with intrahepatic distant recurrence that confirmed single tumor less than 5 cm in size or no more than three tumors, all less than 3 cm in diameter, and Child-Pugh class A or B were included after excluding 574 patients as follows: (1) 321 patients without recurrence; (2) 153 patients with local recurrence; (3) 91 patients with intermediate/ advanced/ terminal stage recurrent HCC; (4) 3 patients with missing or incomplete laboratory data; and (5) 6 patients followed-up with less than 3 months post-procedure. The diagnosis of HCC was made based on the American Association for the Study of Liver Disease (AASLD) practice guidelines [3]. The institutional review board approved the retrospective study and waived the requirement for informed consent.

All clinical and laboratory parameters were collected and reviewed from patient records. The albumin-bilirubin (ALBI) score was computed by the formula, -0.085 x (albumin g/l) + 0.66 x log (bilirubin l mol/l) [19]. ALBI grade was defined by the score of the following: ≤–2.60 = Grade 1, >–2.60 to ≤–1.39 = Grade 2, >–1.39 = Grade 3. ALBI grade 2 was further divided into 2 subgrades (2a and 2b) using a previously reported cut-off value (ALBI score −2.270) and the four ALBI grades were named as modified ALBI grade [20]. End stage renal disease is defined as an estimated glomerular filtration rate less than 15 mL per minute per 1.73 m^2^ body surface area, or those requiring dialysis irrespective of glomerular filtration rate.

### Treatment for recurrent HCC and follow-up

Treatment strategy and decision for IDR was made by a multidisciplinary team according to each individual patient’s clinical status and tumor characteristic. All authors were involved in guiding patient treatment. Under real-time ultrasound guidance, RFA was performed by hepatologists with over five years of experience in ultrasound-guided procedures. The Cool-tip™ RF Ablation System (Medtronic, Minneapolis, MN, USA), Big-tip (RF Medical Co., Seoul, Korea), or Viva RF electrode system (STARmed, Seoul, South Korea) was used, with the algorithm of deposited energy following the manufacturer’s instructions. After completing the RFA procedure, we cauterized the electrode path to avoid bleeding and track seeding of tumor.

For transcatheter arterial chemoembolization (TACE), a microcatheter was catheterized beyond the lobar or segmental branch of the hepatic artery supplying the HCC. Absolute ethanol (99.5%) and lipiodol were mixed in a 3:1 ratio or 20 mg of Epirubicin along with 6ml of lipiodol was administered. The amount of lipiodol used was proportional to the maximum diameter of targeted HCC. If there were multiple HCC, the amount of lipiodol used was adjusted according to the sum of maximum diameters of all targeted HCC in centimeters; however, the maximum amount of lipiodol was limited to 10mL. The TACE would be finished when the infusion of embolizing mixture had been completed or when the retrograde filling of the portal vein was observed.

Contrast-enhanced computed tomography (CECT) or magnetic resonance imaging (MRI) was arranged at one-month post-procedure to evaluate the technical effectiveness of the treatment. The response to TACE was assessed by the Response Evaluation Criteria In Solid Tumors (RECIST) criteria in a CECT scan for a complete, partial or no response. For RFA, the response was assessed by CECT or MRI where complete ablation was defined as the absence of contrast enhancement in the ablated area. Intrahepatic recurrence was defined as a new lesion with arterial contrast enhancement and portal venous washout according to the AASLD guidelines [3]. Intrahepatic distant recurrence and local recurrence were evaluated during follow-up. Local recurrence was defined as the appearance of enhanced tumor around the ablation zone while IDR was defined as appearance of tumor distant to the ablation zone on the basis of standardized terminology and reporting criteria by the International Working Group of Image-Guided Tumor Ablation [21]. The study protocol was approved by the Institutional Review Board of Chang Gung Memorial Hospital, Taiwan.

### Statistical analysis

The categorical variables were expressed as numbers and percentages (%), and continuous variables as mean ± standard deviation (SD). Univariate analysis was performed using the non-paired Student’s *t*-test, Chi-square test or Fisher’s exact test, as appropriate. OS was defined as the period from the date of IDR detection to the date of death related to any cause or the last follow-up until September 2019. The underlying cause of death was classified according to the death certificate data. Subgroup 2^nd^ recurrence analysis was performed for patients who developed second intrahepatic recurrences after complete treatment. The OS and 2^nd^ recurrence rates were measured by using the Kaplan–Meier method and compared using the log-rank test. We analyzed age, gender, HBsAg, Anti-HCV, recurrent tumor number and size, alpha-fetoprotein, modified ALBI score, APRI index, time to 1^st^ recurrence and time to 2^nd^ recurrence, treatment modalities for recurrent HCC as risk factors for OS and 2^nd^ recurrence by univariate and multivariate Cox proportional hazard regression models.

To minimize confounding bias between RFA and TACE groups, a two-to-one, nearest-neighbor matching method using propensity scores generated from logistic regression was performed using R software (Version 3.6.1). The logistic regression was estimated to include age, ESRD, heart failure, total bilirubin level, albumin, prothrombin time, M-ALBI grade, and APRI. All tests for differences were two-tailed, and p-values were considered statistically significant when the associated probability was less than 0.05. Statistical analysis was performed using the SPSS software program (Version 22.0).

## Results

### Overall survival and recurrence

The baseline characteristics of the study patients are shown in Table 1. A total of 349 patients had 463 recurrent HCC. The mean age of the study patients was 66.9 years, with chronic hepatitis B seen in 37% and HCV in 59.6% of the 349 patients. For baseline liver function, 91.1% of patients exhibited Child-Pugh class A. The proportion of diabetic patients was 32.4%, and 8.6% of the patients had ESRD. For tumors, 73.1% of patients had one single tumor only, and 1.7% of the patients had a tumor size larger than 3cm. To treat recurrent HCC, 66.8% of the patients received RFA, 22.6% received TACE, 6.3% received PEI, and 2% received surgery.

**Table 1:** Demographic and clinicopathological features of all intrahepatic distant recurrent patients with hepatocellular carcinoma in early-stage (n = 349).

Variables	Total
Age, years (mean±SD)	66.9 ± 10.0
Gender, male/female(%)	209 (59.9%)/ 140 (40.1%)
HBsAg (positive(%))	129 (37%)
Anti-HCV (positive(%))	208 (59.6%)
Albumin (g/dl)	3.9 ± 0.6
Total bilirubin (mg/dl)	1.0± 0.6
Platelet count (x10^4^/ul)	122.5 ± 59.2
Prothrombin activities (%)	85.6 ± 15.9
Alpha-fetoprotein ≥20 ng/ml (%)	125 (35.8%)
Child-Pugh classification A5(%)/A6(%)/B7(%)	246 (70.5%)/ 72 (20.6%)/ 16 (4.6%)
Recurrent tumor size, cm (mean±SD)	1.8 ± 0.6
Recurrent tumor size, <1cm(%)/1-2cm(%)/2-3cm(%)/>3cm(%)	18 (5.2%)/ 212 (60.7%)/ 103 (29.5%)/ 16 (1.7%)
Recurrent HCC number 1(%)/2(%)/3(%)	255 (73.1%)/ 71 (20.3%)/ 23 (6.6%)
Modified ALBI grade 1(%)/ 2a(%)/ 2b(%)/ 3(%)	169 (48.1%)/ 63 (18.1%)/ 101 (28.9%)/ 11 (3.2%)
BCLC staging 0(%)/A(%)	189 (54.2%)/ 160 (45.8%)
AJCC 8^th^ TNM staging IA(%)/IB(%)/II(%)	190 (54.4%)/ 62 (17.8%)/ 97 (27.8%)
Treatment for recurrence	
RFA	233 (66.8%)
PEI	22 (6.3%)
TACE	81 (23.2%)
RFA & TAE	4 (1.1%)
Surgery	7 (2%)
No treatment	2 (0.6%)

Data are expressed as mean ±standard error of mean or number (percentage).

Abbreviations: AJCC, American Joint Committee on Cancer; ALBI grade, Albumin-Bilirubin grade; Anti-HCV, Anti-hepatitis C virus antibody; BCLC, Barcelona clinic liver cancer; HBsAg, Hepatitis B virus surface antigen; HCC, hepatocellular carcinoma; PEI, Percutaneous ethanol injection; RFA, radiofrequency ablation; TACE, Transcatheter arterial chemoembolization.

**Fig 2:**
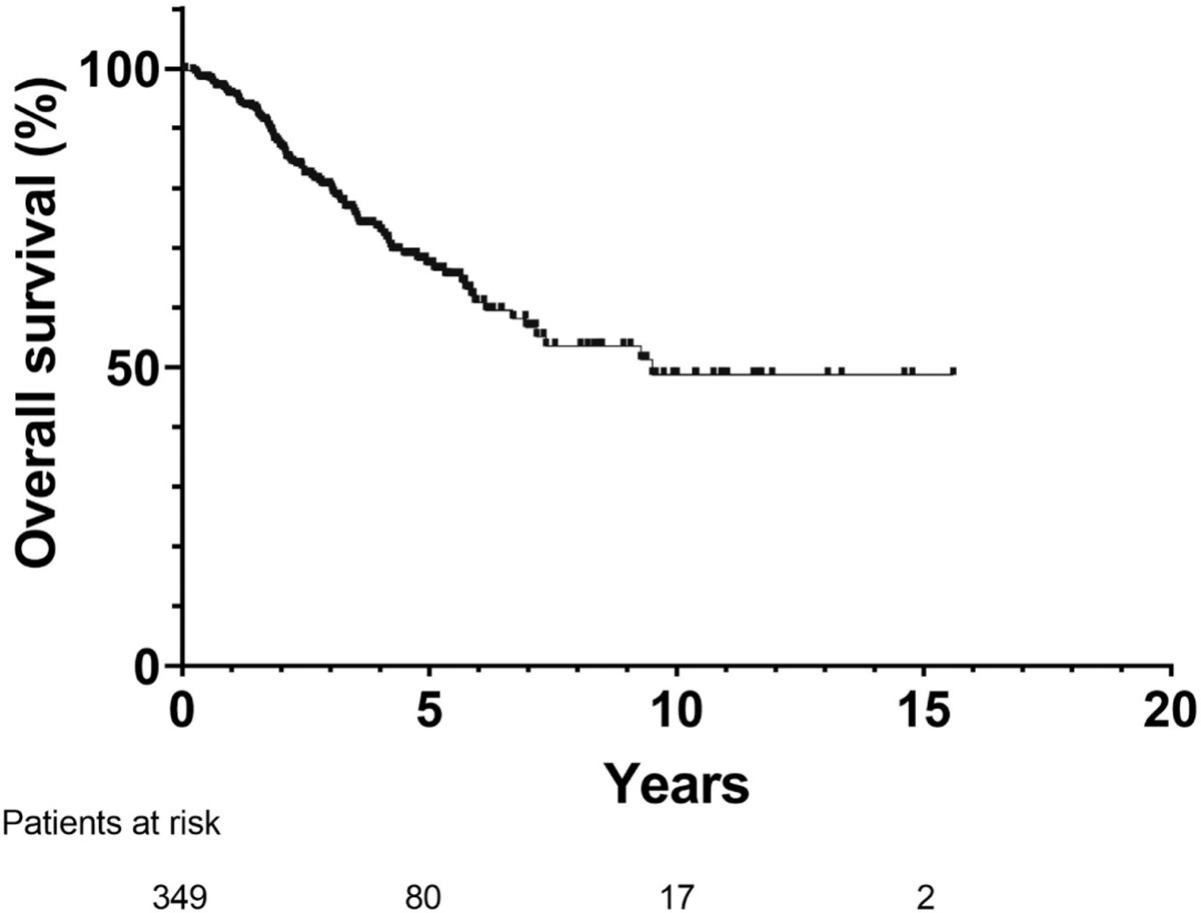
Overall survival of 349 patients with early-stage recurrent hepatocellular carcinoma. The 1-, 3-, and 5-year overall survival rates were 95.5%, 80.4%, and 67.2% respectively.

At the end of follow-up, a total of 92 patients had expired. The median follow-up time was 3.02 years. The 1-, 3- and 5-year cumulative OS rates were 95.5%, 80.4% and 67.2% respectively (Fig 2). Complete treatment for IDR was achieved in 317 of the 345 patients (91.8%) and 2^nd^ tumor recurrence occurred in 242 patients (76.3%) over a median follow-up of 1.2 years.

**Fig 3:**
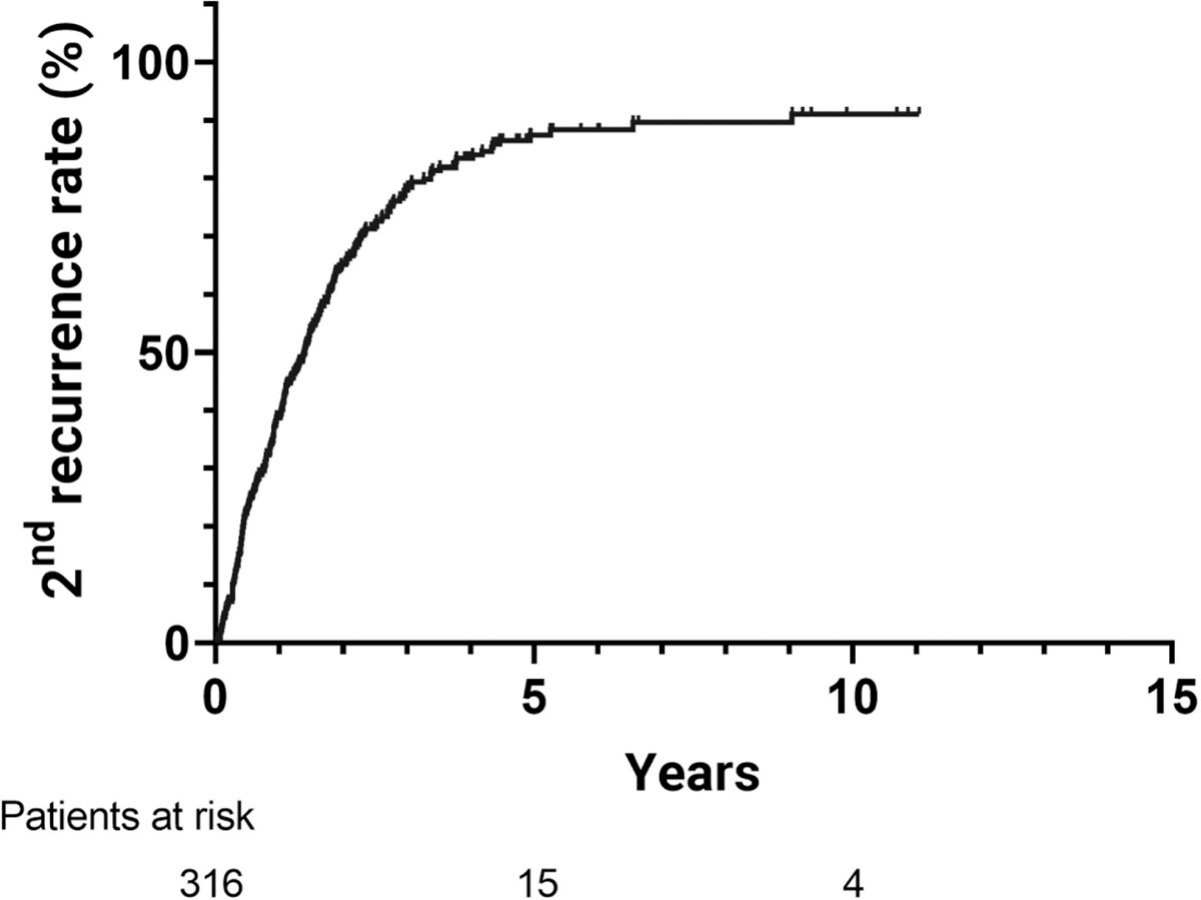
Overall 2nd recurrence rates of patients with early-stage IDR after complete treatment. The 1-, 3-, and 5-year 2nd overall recurrence rates after complete treatment was 38.8%, 77.9%, and 87.4% respectively. IDR, intrahepatic distant recurrence.

**Fig 4:**
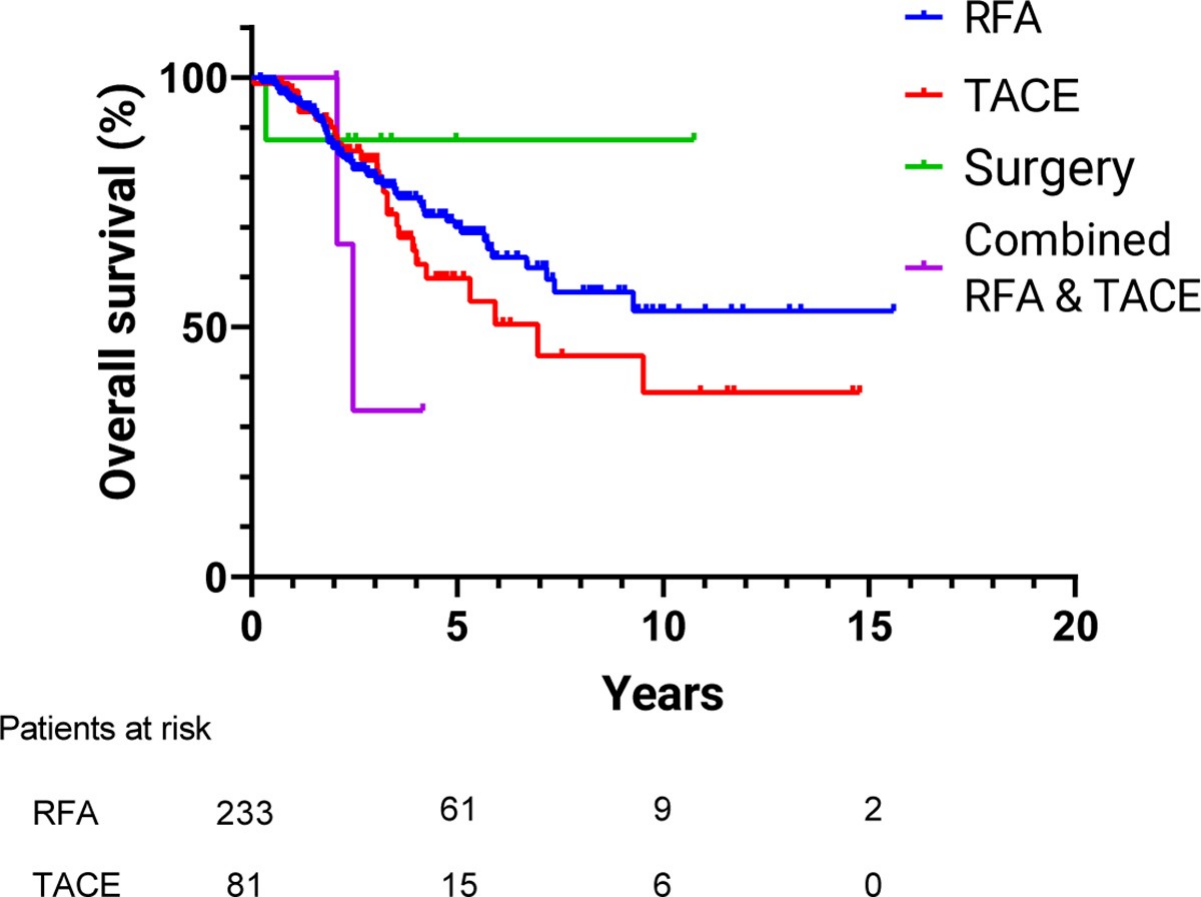
Cumulative overall survival of different treatment groups with IDR. The overall survival rates at 1, 3, and 5 years of patient treatment with RFA for IDR (95.5%, 80.4%, and 70.1% respectively) were similar to those treated with TACE for IDR (97.4%, 83.5%, and 59.8% respectively; p = 0.257). IDR, intrahepatic distant recurrence; RFA, radiofrequency ablation; TACE, Transcatheter arterial chemoembolization.

**Fig 5:**
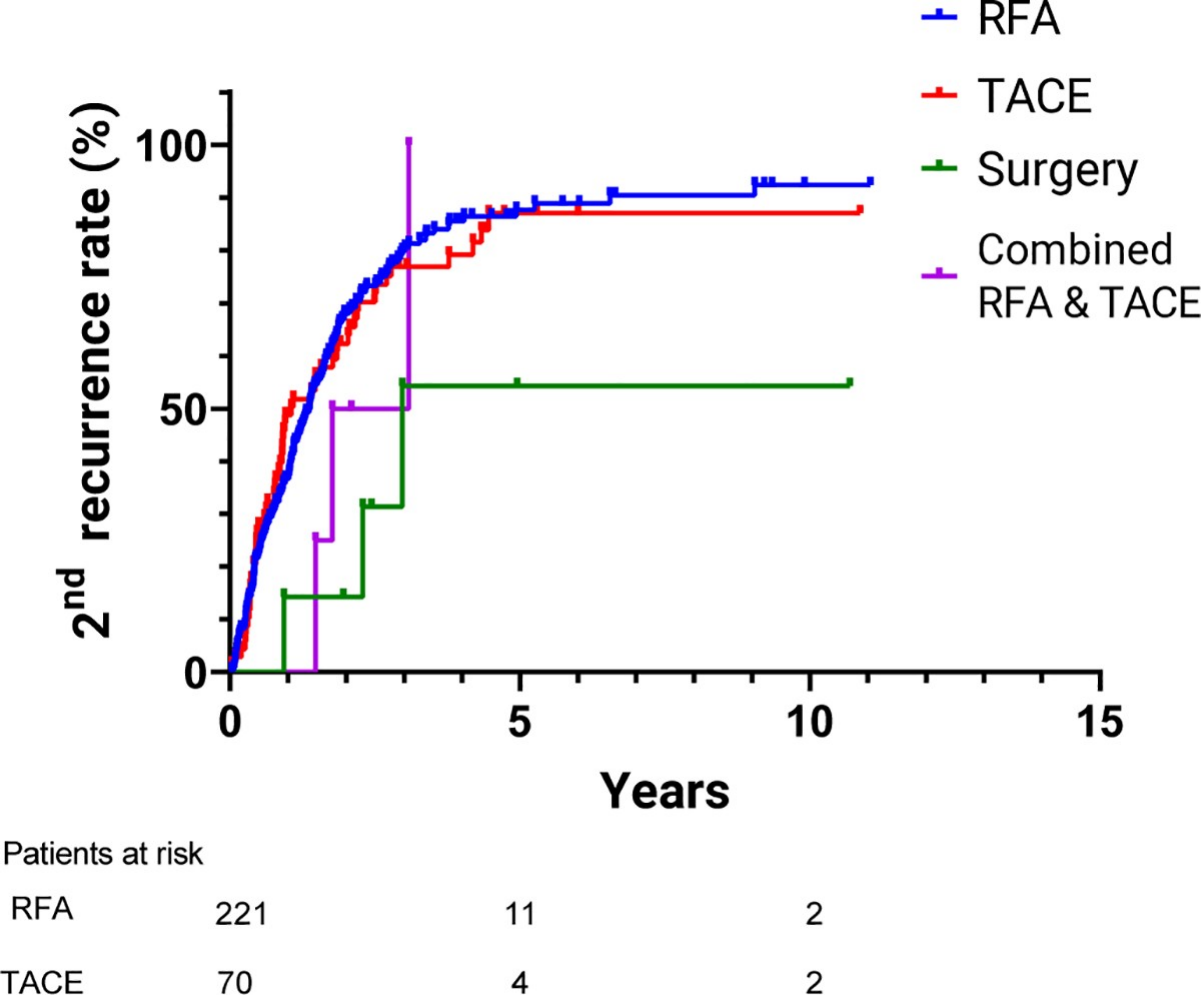
Cumulative 2nd recurrence rates of different treatment groups with IDR. The cumulative 1-, 3- and 5-year 2nd recurrence rates were 36.8%, 79.8% and 87.7% respectively in the RFA group, and 48.8%, 76.9% and 87.2% respectively in the TACE group. There was no significant difference in 2nd recurrence rate between RFA and TACE group (p = 0.86). RFA, radiofrequency ablation; TACE, Transcatheter arterial chemoembolization.

The 1-, 3- and 5-year cumulative 2^nd^ overall recurrence-free rates after treatment were 61.1%, 22.1% and 11.7% respectively (Fig 3). Of all the 2^nd^ recurrences, 52.5% were IDR, 35.1% were local recurrence, and 12.4% were concurrent local and IDR. Among the patients who received RFA for IDR, the 1-, 3-, and 5-year OS rates were 95.5%, 80.4% and 70.1% respectively, while cumulative 2^nd^ recurrence rates after treatment were 36.8%, 79.8% and 87.1% respectively. In addition, for the patients who received TACE for IDR, the 1-, 3- and 5-year OS rates were 97.4%, 83.5% and 59.8% respectively, while the cumulative 2^nd^ recurrence rates after treatment were 48.8%, 76.9% and 87.2% respectively (Figs 4 and 5). Comparing the outcomes between patients receiving RFA and TACE for IDR, there was no significant difference in the OS (TACE vs. RFA H.R. = 1.31, 95% confidence interval (CI) (0.82–2.10), p = 0.26) and 2^nd^ recurrence rate (TACE vs. RFA H.R. = 0.97, 95% confidence interval (CI) (0.71–1.31), p = 0.83), and the recurrence pattern between the two treatment modalities were similar.

To balance the clinical variables between RFA and TACE groups, a total of 213 patients (142 from RFA group; 71 from TACE group) were matched by applying two-to-one propensity score matching. Matched pairs showed similar baseline characteristics (Table 2). In the matched cohort, the 1-, 3- and 5-year OS rates were 94.8%, 77.1%, and 65.1% respectively in the RFA group, and 98.6%, 84.4%, and 58.6% respectively in the TACE group; a difference that was not significant (p = 0.50). In addition, the 2^nd^ recurrence rates did not differ between the two groups after propensity score matching. (38.9% vs. 48.6% at 1 year; 78.4% vs. 76.5% at 3 years; 84.3% vs. 85.4% at 5 years; p = 0.89).

**Table 2:** Demographic and clinicopathological features of the RFA and TACE group after propensity score matching.

Variable	RFA (N = 142)	TACE (N = 71)	P value
Age, years (mean ±SD)	67.3±10.6	67.1±10.1	0.94
Sex (male)	82 (57.7%)	45 (63.4%)	0.43
HBsAg (+)	46 (32.5%)	23 (32.4%)	>0.99
Anti-HCV (+)	91 (64.1%)	47 (66.7%)	0.88
End-stage renal disease	8 (5.6%)	4 (5.6%)	>0.99
Heart failure	20 (14.1%)	11 (15.5%)	0.95
Child Pugh class A (%)	131 (92.3%)	68 (95.8%)	0.49
Albumin (g/dl)	3.9±0.6	3.9±0.5	0.86
Total bilirubin (mg/dl)	1.0±0.5	1.0±0.5	0.96
Prothrombin activities (%)	85.5±16.5	84.0±14.2	0.76
α-fetoprotein >20ug/L	61 (43%)	31 (43.7%)	0.92
Recurrent HCC number 1(%)/ 2(%)/ 3(%)	103 (57.7%)/ 29 (20.4%)/ 10 (7%)	49 (69%)/ 12 (16.9%)/ 10 (14.1%)	0.25
Recurrent HCC size, cm (mean ±SD)	1.8±0.6	1.8±0.7	0.64
Modified-ALBI 1/ 2a/ 2b+3	67 (47.2%)/ 32 (22.5%)/ 43 (30.3%)	30 (42.3%)/ 18 (25.4%)/ 23 (32.4%)	0.79
APRI >1	89 (62.7%)	46 (64.8%)	0.88

Data are expressed as mean ±standard error of mean or number (percentage).

Abbreviations: ALBI grade, Albumin-Bilirubin grade; Anti-HCV, Anti-hepatitis C virus antibody; APRI, aspartate aminotransferase to platelet ratio index; HBsAg, Hepatitis B virus surface antigen; HCC, hepatocellular carcinoma; RFA, radiofrequency ablation; TACE, Transcatheter arterial chemoembolization.

### Factors associated with overall survival and recurrence

**Fig 6:**
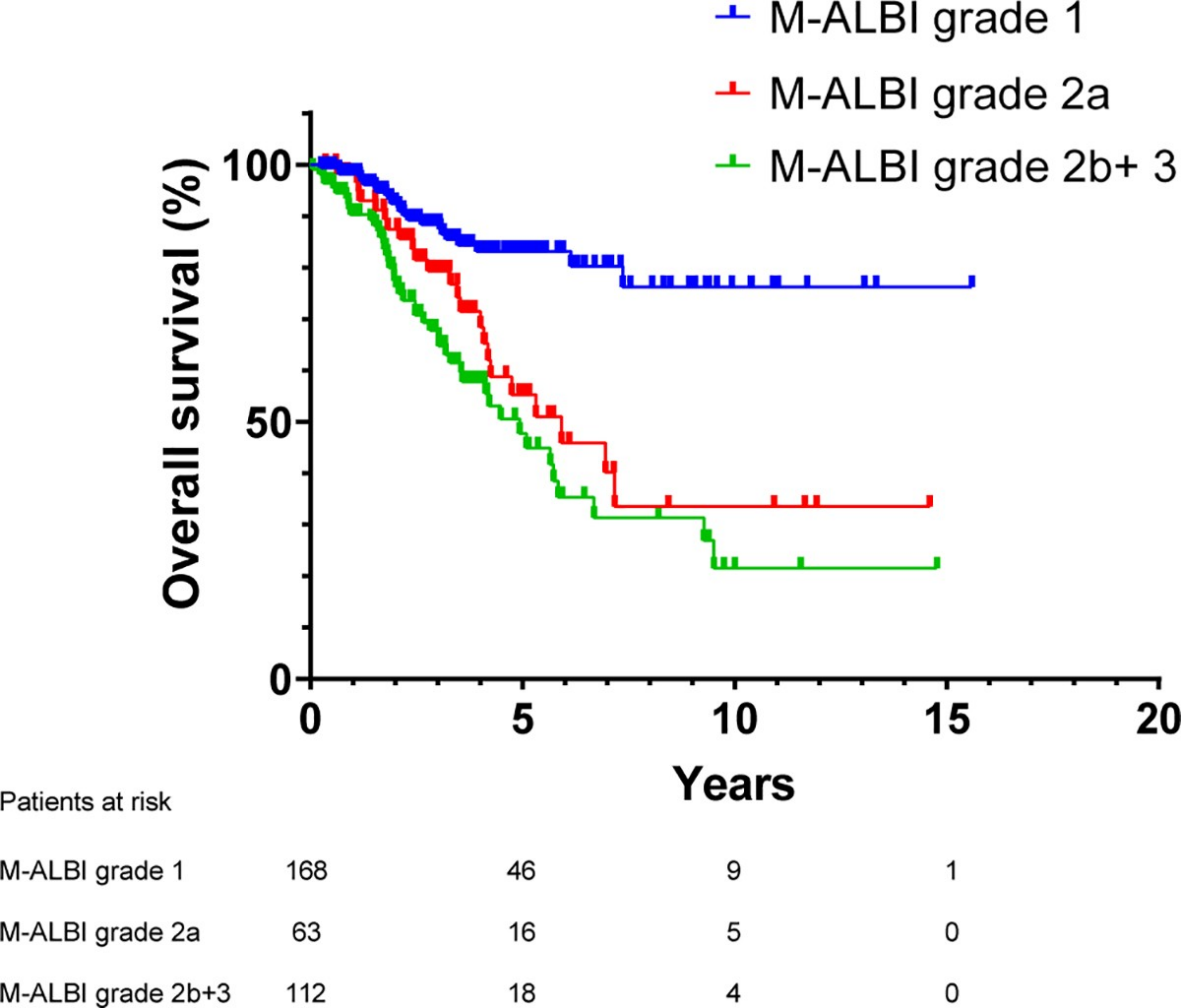
Overall survival in patients with intrahepatic distant recurrence. Patients with m-ALBI grade 1 had better OS than m-ALBI grade 2a (p = 0.001) and m-ALBI grade 2b+3 (p<0.001). M-ALBI grade, modified albumin-bilirubin grade.

The factors associated with poor OS were assessed by using univariate and multivariate analysis (Table 3). Multivariate analysis showed that ESRD (H.R. = 2.33, 95% CI (1.14–4.80), p = 0.021), m-ALBI grade 2a (H.R. = 2.86, 95% CI (1.45–5.67), p = 0.003) and grade 2b or 3 (H.R. = 2.30, 95% CI(1.25–4.68), p = 0.009) (Fig 6), APRI greater than 1 (H.R. = 1.92, 95% CI(1.05–3.52), p = 0.036) and 2nd recurrence occurring within 1 year (H.R. = 2.69, 95% CI(1.61–4.49), p<0.001) were significantly associated with worse OS.

**Table 3:** Univariate and multivariate analysis of the factors associated with overall survival.

Variable	Univariate analysis			Multivariate analysis			
	HR	(95% CI)	P value	HR	(95% CI)		P value
Sex (male vs. female)	1.041	(0.688–1.574)	0.851				
HBsAg (+ vs. -)	0.864	(0.566–1.320)	0.499				
Anti-HCV (+ vs. -)	0.962	(0.636–1.456)	0.855				
Diabetes mellitus (+ vs. -)	1.102	(0.709–1.714)	0.665				
End-stage renal disease (+ vs. -)	1.962	(1.089–3.536)	0.025*	2.334	(1.135–4.799)	0.021*	
Heart failure (+ vs. -)	1.759	(1.038–2.980)	0.036*				
Age (> 65 vs. ≤65 years)	2.029	(1.307–3.150)	0.002*				
Alpha fetoprotein (> 20 vs. ≤20 ng/ml)	1.552	(1.026–2.348)	0.037*				
Child-Pugh classification (B vs. A)	1.967	(1.069–3.621)	0.030				
International normalized ratio (>1.1 vs. ≤1.1)	1.775	(1.115–2.728)	0.009*				
Platelet count (<150,000/ul vs. ≥ 150,000/l)	2.381	(1.368–4.143)	0.002*				
Modified ALBI grade							
1		1					
2a	2.785	(1.562–4.966)	0.001	2.864	(1.447–5.669)	0.003*	
2b and 3	3.980	(2.405–6.590)	<0.001	2.298	(1.251–4.680)	0.009*	
APRI index (>1 vs. ≤1)	2.563	(1.586–4.140)	<0.001	1.919	(1.045–3.524)	0.036*	
Recurrent tumor number (2 and 3 vs. 1)	0.802	(0.484–1.329)	0.391				
Recurrent tumor size (> 2 vs. ≤2cm)	0.879	(0.544–1.419)	0.597				
Received RFA for recurrence (+ vs. -)	0.806	(0.501–1.296)	0.373				
Received TACE for recurrence (+ vs. -)	1.379	(0.882–2.156)	0.159				
Received Surgical intervention for recurrence (+ vs. -)	0.232	(0.032–1.669)	0.147				
2nd recurrence (+ vs. -)	1.456	(0.786–2.696)	0.233				
Time to 1st recurrence (<2 vs. ≥2 year)	1.762	(1.090–2.847)	0.021*				
Time to 2nd recurrence (<1 vs. ≥1year)	2.338	(1.436–3.808)	0.001*	2.685	(1.605–4.492)	<0.001*	

Abbreviations: APRI, aspartate aminotransferase to platelet ratio index; CI, confidence interval; HR, hazard ratio; modified-ALBI grade, modified albumin-bilirubin grade; RFA, radiofrequency ablation; TACE, transcatheter arterial chemoembolization.

Table 4 shows the results of univariate and multivariate analysis of risk factors associated with 2nd HCC recurrence after treatment. With respect to the 2^nd^ recurrence after treatment of the IDR, male sex (H.R. = 1.47, 95% CI(1.10–1.97), p = 0.01), age greater than 65 years (H.R. = 1.73, 95% CI(1.28–2.30), p = 0.006), an AFP level greater than 20 ng/ml (H.R. = 1.52, 95% CI(1.14–2.04), p = 0.004), tumor number greater than 1 (H.R. = 1.35, 95% CI(1.01–1.81), p = 0.046), and IDR developing within 2 years (H.R. = 1.66, 95% CI(1.23–2.24), p = 0.001) were associated with higher risks of 2^nd^ recurrence while surgical treatment for IDR resulted in lower risk of 2^nd^ recurrence (H.R. = 0.25, 95% CI(0.09–0.68), p = 0.007).

**Table 4:** Univariate and multivariate analysis of the factors associated with 2nd recurrence.

Variable	Univariate analysis			Multivariate analysis		
	HR	(95% CI)	P value	HR	(95% CI)	P value
Sex (male vs. female)	0.862	(0.667–1.116)	0.260	1.470	(1.099–1.968)	0.010*
HBsAg (+ vs. -)	0.782	(0.598–1.023)	0.072			
Anti-HCV (+ vs. -)	1.079	(0.833–1.398)	0.565			
Diabetes mellitus (+ vs. -)	0.908	(0.688–1,197)	0.492			
End stage renal disease (+ vs. -)	1.016	(0.649–1.591)	0.943			
Age (> 65 vs ≤65 years)	1.500	(1.150–1.957)	0.003*	1.716	(1.280–2.300)	<0.001*
Alpha fetoprotein (> 20 vs. ≤20 ng/ml)	1.529	(1.176–1.989)	0.002*	1.405	(1.066–1.852)	0.016*
Child-Pugh classification (B vs. A)	1.133	(0.717–1.793)	0.592			
International normalized ratio (>1.1 vs. ≤1.1)	1.069	(0.796–1.436)	0.657			
Platelet count (<150,000/ul vs. ≥150,000/ul)	1.442	(1.084–1.918)	0.012*			
Modified-ALBI grade						
1		1				
2a	1.316	(0.938–1.838)	0.113			
2b and 3	1.588	(1.186–2.126)	0.002*			
APRI index (>1 vs. ≤1)	1.387	(1.062–1.811)	0.016*			
Recurrent tumor number (2 and 3 vs. 1)	1.405	(1.066–1.853)	0.016*	1.350	(1.005–1.814)	0.046*
Recurrent tumor size (> 3 vs. ≤3 cm)	0.557	(0.207–1.499)	0.247			
Received RFA for recurrence (+ vs. -)	1.074	(0.785–1.471)	0.654			
Received TACE for recurrence (+ vs. -)	0.962	(0.719–1.286)	0.793			
Received Surgical intervention for recurrence (+ vs. -)	0.239	(0.089–0.645)	0.005*	0.250	(0.092–0.681)	0.007*
Time to 1st recurrence (<2 vs. ≥2 year)	1.765	(1.342–2.323)	<0.001*	1.659	(1.230–2.238)	0.001*

Abbreviations: APRI, aspartate aminotransferase to platelet ratio index; CI, confidence interval; HR, hazard ratio; modified-ALBI grade, modified albumin-bilirubin grade; RFA, radiofrequency ablation; TACE, transcatheter arterial chemoembolization

## Discussion

Nonlocal intrahepatic recurrence of HCC after RFA for early-stage HCC is reported to be 70–76% by 5 years [9, 18]. In our study, the 5-year IDR rate after RFA for primary HCC was 71.2%, and this result is consistent with those of previous studies. The treatment algorithm for recurrent HCC after RFA has not been established in guidelines. Salvage liver transplantation and hepatectomy have been reported to be the optimal treatment but is limited by organ shortage, tumor multiplicity and the operation is technical demanding [22–24]. RFA has been accepted as treatment for patients who are not eligible for further hepatectomy after curative treatment. Also, TACE is often selected because of the presence of multiple nodules, poor conspicuity by ultrasound and the unfavorable nodule location for RFA. In our patients with IDR, only three patients received transplantation and four patients received salvage hepatectomy, two-thirds received RFA, 22.5% received TACE and 1.1% received combination of RFA and TACE for the 1^st^ recurrent HCC. The 5-year OS of patients with IDR was 67.2%, which is consistent with the result of previous studies [9, 18]. In the subgroup who received RFA for recurrent HCC, 94.5% of the patients achieved complete response, and the 5-year OS and recurrence rates were 70.1% and 88% respectively. The result of OS is comparable to that for primary HCC but the recurrence rate is higher than that for primary HCC [7, 11, 25]. Fukuhara et al. compared RFA for recurrent HCC after curative treatment with RFA for primary cases, and OS was similar between the two groups while disease-free survival was shorter in the recurrent group, which is consistent with our finding [25]. Rossi et al. reported one out of three patients developed some type of nonlocal recurrence each year and over 65% of recurrent episodes were managed with repeat RFA with 96.5% achieving complete response [26]. Our study echoes that high repeatability of RFA and its value for controlling intrahepatic recurrences.

The difference in OS between different treatment modalities was insignificant in our study yet the concurrent RFA with the TACE group revealed a trend of inferior outcome (Fig 3). After propensity score-matching to the RFA and TACE group, the difference of OS between these two groups remained insignificant. Okuwaki et al. compared OS between RFA and TACE for single, small IDR and their study showed that repeat RFA for IDR was the only significant favorable prognostic factor [18].

As 76.6% of the patients in our study experienced 2^nd^ recurrent HCC and most patients received multiple and different subsequent treatments, this might result in insignificant difference between different treatment groups for 1^st^ recurrence. In addition, there were only seven and four patients in the surgery group and combination RFA and TACE group respectively, probably resulting in insufficient statistical power.

We analyzed clinical data when HCC recurrence occurred and identified poor liver function reserve assessed by m-ALBI and APRI, presence of ESRD, and time to 2^nd^ recurrence developing within 1 year were associated with poor outcome.

IDR might represent either intrahepatic metastases or development of de novo tumors [27]. Previous studies have indicated OS is related to early recurrence [28], and a cutoff of 2 years has been generally adopted to classify early and late recurrence [4]. Our study discloses that once IDR has developed, the interval of 2^nd^ recurrence occurring within 1 year is a prognostic factor for OS, while the interval of 1^st^ recurrence is associated with higher risk of 2^nd^ recurrence but not to OS.

Previous studies have shown poor liver function reserve is associated with poor OS after RFA for HCC. The Child-Pugh grade has been incorporated in many HCC classifications and extensively used to evaluate liver reserve in patients with liver cirrhosis, but grade of ascites and encephalopathy are subjective parameters and 20% of patient with HCC do not have cirrhosis. The ALBI grade has been proven to be as effective as the Child-Pugh grade, is an independent prognostic factor of outcome for patients with early HCC after RFA [12], and for patients with recurrent HCC after hepatectomy as well [29]. Modified ALBI grade (m-ALBI) divides ALBI score into four groups and has been proven to have fair stratification ability and prognostic predictive value in patients with HCC [14, 30]. As liver function reserve might change after RFA, we used m-ALBI grade to evaluate patient’s liver function reserve at recurrence. The OS of patients in the m-ALBI grade 1 group was superior to other groups (Fig 6); however, the difference of OS between m-ALBI grades 2a and 2b+3 was insignificant. The possible explanation to the result is that 64.3% of patients in m-ALBI grade 2b/3 group developed 2nd recurrence within 1 year while the proportion is 35.7% in m-ALBI grade 2a group. Therefore, the difference of overall survival between these two groups would be attenuated when the factor of 2nd recurrence develop within 1 year is adjusted.

APRI has been validated as a simple, non-invasive predictor for assessing the degree of fibrosis in chronic hepatitis C [31, 32]. In the study of Aye et al., APRI was well performing in predicting significant fibrosis in patients with chronic hepatitis B as well [33]. Paik et al. reported APRI and FIB-4 could stratify HCC risk for chronic HBV-infected patients [34], and Hann et al. indicated APRI might be a marker of HCC risk in HBV patients in cirrhosis-dependent and -independent manners [35]. In addition, APRI is associated with OS and recurrence for patients with HCC after RFA therapy [15, 36]. This study demonstrates that the discriminative ability of APRI with cutoff value 1 remains good for OS after intrahepatic HCC recurrence has occurred.

As comorbidity is more prevalent in older patients, we analyzed several comorbidities that might affect OS and found that ESRD is related to worse outcome when recurrence of HCC is present. In our study cohort, there were thirty patients with end stage renal disease and twelve of them had died at the end of follow-up. The cause of death could be attributed to HCC in progression or complication of cirrhosis such as variceal bleeding and hepatic encephalopathy in seven patients. The rest of the patients died of non-liver related cause such as myocardial infarction and intracranial hemorrhage. Previous studies have demonstrated HCC patients on hemodialysis had a 2.036-fold greater chance of death than HCC patients not on hemodialysis [37] and ESRD has been found to be a predictor of mortality and hemorrhagic complications [38].

The present study identified male gender, age> 65 years, AFP> 20ng/ml, multiple recurrent tumors, and 1^st^ recurrence occurring within 2 years bear higher risks of 2^nd^ recurrence after treatment for recurrent HCC. On the contrary, patients who received surgical intervention for recurrent HCC have lower risk of 2^nd^ HCC recurrence. In the predictive model proposed by Chan et al., male gender is related to early recurrence of HCC after surgical resection [39]. In another multicenter study from China, male sex was a risk factor of late recurrence after liver resection for HCC as well [40]. In our study, male sex was associated with higher risk of 2^nd^ recurrence after complete treatment for IDR. A recent meta-analysis showed no significant difference in OS between RFA and surgical resection for intrahepatic HCC recurrence; however, surgical resection was associated with higher 2^nd^ recurrence-free survival, which is similar to our findings [41].

Multinodularity and tumor size are both associated with the prognosis for patients with HCC undergoing RFA [42]. Nishikawa et al. reviewed patients who received RFA for single HCC and those with single intrahepatic recurrence had similar OS as those without recurrence, yet the patients with two or three recurrent tumors had worse OS [43]. In the present study, multinodularity was associated with higher risk of 2^nd^ recurrence but no tumor factor was associated with OS after the appearance of IDR, which might be due to our patient groups being confined to early- and very early-stage intrahepatic recurrence while only 4.5% of patients had tumor size >3cm.

There are several limitations in our study. Firstly, incomplete or missing clinical data are inevitable when retrospectively reviewing medical records. Secondly, the patients were recruited from a single medical center; however, referral is not mandatory under the present healthcare system in Taiwan so our patients could be closer to general HCC populations. Thirdly, our analysis lacked antiviral therapy and tumor recurrence site, which might affect prognosis. Fourthly, the number of cases in the surgical group was small, so further analyses including randomized controlled trials are warranted to clarify whether repeat RFA is the optimal treatment for IDR.

## Conclusions

The discriminative ability of ALBI grade and APRI for OS in recurrent (very) early- stage HCC remains reliable. Other prognostic factors of OS include ESRD and further recurrence developing within 1 year. Old age, male gender, high AFP level, multiple recurrent tumors, and early IDR within 2 years would result in higher risk of 2^nd^ recurrence, while surgical intervention could decrease the likelihood of 2^nd^ recurrence.

## Supporting information

S1 Data (XLSX)
